# Supplementary material for: A Convenient Plant-Based Detection System to Monitor Androgenic Compound in the Environment
Source: Plants (Basel). 2019 Aug 5;8(8):266. doi: 10.3390/plants8080266 (PMC6724103; doi:10.3390/plants8080266)
Supplement: Supplementary file 1 [file plants-08-00266-s001.pdf]

Table S1. List of the primer sequences used in this study

name	Locus/ Template	Primer Sequences	purpose
AtACT2	AT3G18780	F: 5′-GCAGAGCGGGAAATTGTAAG-3′<br>R: 5′-TTCTCGATGGAAGAGCTGGT-3′	qRT-PCR
AtCHS	AT5G13930	F: 5′-ACGTCACGTGTTGAGCGAGT-3′<br>R: 5′-CCACTCCAACCCTTCTCCTG-3′	qRT-PCR
AtCHI	AT3G55120	F: 5′-TCTCTCCCCTACCGGCTCTC-3′<br>R: 5′-ACACACCGTTCTTCCCGATG-3′	qRT-PCR
AtF3H	AT3G51240	F: 5′-GAGGAGCCAATCACGTTTGC-3′<br>R: 5′-GCGAAGATTTGGTCGACAGG-3′	qRT-PCR
AtDFR	AT5G42800	F: 5′-AGGGTTTTCTCCCGGTTTCA-3′<br>R: 5′-CCGGTTATCCCCGTTTCTGT-3′	qRT-PCR
AtANS	AT4G22880	F: 5′-CTTTGGATTGGGGAGTGATGC-3′<br>R: 5′-GTCCACTCGCGTTGTTAGCC-3′	qRT-PCR
AtUFGT	AT5G17050	F: 5′-CTTCAACACCGCACAATCCA-3′<br>R: 5′-TCCTGTGGTCTCCCGCTAAA-3′	qRT-PCR
PtrMYB119	Potri.017 G125600	F: 5′-TCCTTCCAGAGCAGGCTTGA-3′<br>R: 5′-CCACTTCGTCCACCGAAAAC-3′	qRT-PCR
XV_SpeI_F	pMDC7	F: 5’-TTTACTAGTATGAAAGCGTTAACGGCCAG-3’	cloning
XV_pA_R	pMDC7	R: 5’-TCGGGCTGGTTGTTagacggatccccaccgtactcgtc-3’	cloning
pXV_A_F	NM_000044.2	F: 5’-gtggggatccgtctAACAACCAGCCCGACTCCTTTGCA-3’	cloning
A_SpeI_R	NM_000044.2	R: 5’-TTTACTAGTTCACTGGGTGTGGAAATAGATG-3’	cloning
